# Simulation of Near-Infrared Light Absorption Considering Individual Head and Prefrontal Cortex Anatomy: Implications for Optical Neuroimaging

**DOI:** 10.1371/journal.pone.0026377

**Published:** 2011-10-24

**Authors:** Florian B. Haeussinger, Sebastian Heinzel, Tim Hahn, Martin Schecklmann, Ann-Christine Ehlis, Andreas J. Fallgatter

**Affiliations:** 1 Department of Psychiatry and Psychotherapy, University of Tuebingen, Tuebingen, Germany; 2 Department of Psychiatry, Psychosomatics and Psychotherapy, University of Wuerzburg, Wuerzburg, Germany; 3 German Center for Neurodegenerative Diseases (DZNE), Bonn, Germany; 4 Department of Cognitive Psychology II, University of Frankfurt am Main, Frankfurt am Main, Germany; 5 Department of Psychiatry and Psychotherapy, University of Regensburg, Regensburg, Germany; Chiba University Center for Forensic Mental Health, Japan

## Abstract

Functional near-infrared spectroscopy (fNIRS) is an established optical neuroimaging method for measuring functional hemodynamic responses to infer neural activation. However, the impact of individual anatomy on the sensitivity of fNIRS measuring hemodynamics within cortical gray matter is still unknown. By means of Monte Carlo simulations and structural MRI of 23 healthy subjects (mean age: 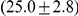 years), we characterized the individual distribution of tissue-specific NIR-light absorption underneath 24 prefrontal fNIRS channels. We, thereby, investigated the impact of scalp-cortex distance (SCD), frontal sinus volume as well as sulcal morphology on gray matter volumes (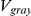) traversed by NIR-light, i.e. anatomy-dependent fNIRS sensitivity. The NIR-light absorption between optodes was distributed describing a rotational ellipsoid with a mean penetration depth of 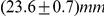 considering the deepest 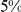 of light. Of the detected photon packages scalp and bone absorbed 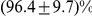 and 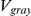 absorbed 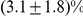 of the energy. The mean 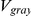 volume 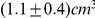 was negatively correlated (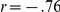) with the SCD and frontal sinus volume (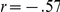) and was reduced by 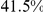 in subjects with relatively large compared to small frontal sinus. Head circumference was significantly positively correlated with the mean SCD (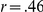) and the traversed frontal sinus volume (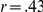). Sulcal morphology had no significant impact on 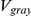. Our findings suggest to consider individual SCD and frontal sinus volume as anatomical factors impacting fNIRS sensitivity. Head circumference may represent a practical measure to partly control for these sources of error variance.

## Introduction

Functional near-infrared spectroscopy (fNIRS) is an optical neuroimaging technique increasingly used to investigate neural processing in healthy subjects and in psychiatric patients [1]. fNIRS relies on the differential absorption of near-infrared (NIR) light by oxygenated and deoxygenated hemoglobin (O2-Hb; H-Hb) and the change in the concentration of O2-Hb and H-Hb mediated by the neurovascular coupling following neural activation. However, other molecules in biological tissue such as melanin also absorb NIR-light [2], but since their concentrations are constant the relative change in NIR-light absorbance during the hemodynamic responses is not impacted by these chromophores [3]. However, due to the highly scattering properties of the heterogeneous biological tissues, a plethora of paths of the emitted NIR-light are possible. As a consequence of NIR-light scattering, only about 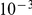 to 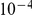 of the light reaches the detector, which is commonly positioned in a distance of 3 cm on the head surface of adults. The average path of the emitted NIR-light has been reported to describe a “banana-shaped” [4], [5] or ellipsoid path [6] with an approximate head penetration depth of about 2 cm to 3 cm [4], [6]. Importantly, fNIRS is a reliable technique [7]–[9] to indirectly detect and quantify neural activity by measuring changes in NIR-light absorption due to O2-Hb and H-Hb concentration changes [3], [10]. Neural signals are mainly integrated, processed and reliably coupled to functional hemodynamic responses within gray matter. Thus, functional neuroimaging commonly focusses on this tissue type.

NIR-light penetration depth has been shown to depend on the scalp-cortex distance (SCD) using spatial distributions of correlations between hemodynamic responses concurrently measured with fNIRS and functional magnetic resonance imaging (fMRI) [6]. However, an impact of individual anatomy and tissue composition on the traversed gray matter volume and its variability between subjects as well as fNIRS-channels, has not been investigated yet. Moreover, while fNIRS measurements of functional hemodynamics within anterior orbitofrontal cortex (OFC) have been conducted [11], the feasibility of fNIRS to measure functional hemodynamics within gray matter underneath frontal sinus sites is still unclear.

The frontal sinus is a fronto-polar air-filled cavity, whose morphology is highly inter-individually variable and may differ among races (mean volume standard deviation (SD): (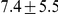) cm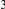, assessed in adult Caucasians [12]; (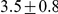) cm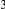 assessed in (young) adult Asians [13]). The frontal sinus is mostly located rostrally to parts of the OFC. Since air-filled frontal sinus has previously been suggested to affect NIR-light propagation [14], [15], we systematically assessed the relationship between frontal sinus volume and NIR-light penetration depth as well as gray matter volume absorbing NIR-light.

In order to provide a numerical solution for the forward problem of photon migration in highly scattering biological tissues, we implemented a Monte Carlo simulation (MCS) of a large number of photon packages. MCS have repeatedly been used to describe characteristics of photon scattering and migration using head or tissue layer models [16]–[19]. Also, MCS has been applied to segmented MRI data to compute fNIRS sensitivity spread functions within the visual cortex [20]–[22]. Hitherto, a MCS of spatial tissue-specific distributions of NIR-light absorbance in the prefrontal cortex has not been conducted.

The MCS implemented in our study considered the following basic principles. The light attenuation between an emitter-detector pair is decribed by 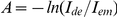 where 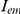 is the intensity of light eradiated by the emitter and 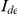 is the intensity measured by the detector. The rate of absorption and scattering of a certain tissue 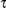 depends on the absorption coefficient 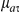 and the scattering coefficient 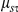. Derived from the *Radiation Transport Equation* the *Beer-Lambert* law can be used to calculate the attenuation 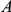 between arbitrarily arranged optodes in an optically homogeneous medium (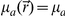) [23],
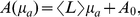
(1)where 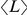 is the mean pathlength of all photons and 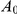 is the fraction of attenuation solely caused by scattering and not by absorption [24]. The total attenuation of light traveling through different homogeneous tissue types, can be calculated by the sum of the partial attenuations. The differential of the total attenuation over 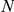 (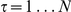) tissue types is proprotional to fNIRS signal changes during a measurement and can be described by:
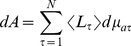
(2)Neural activity is followed by a local increase in blood flow, predominantly in gray matter, which relatively increases 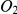-Hb and decreases H-Hb concentrations in the blood, thereby changing the absorption coefficient of a given wavelength and thereby the NIR-light attenuation 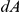. However, 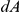 is proportional to the mean pathlength of the light 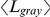 through gray matter. Thus, the sensitivity of fNIRS to detect changes in 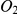-Hb and H-Hb concentrations directly depends on the traversed gray matter volume and the mean partial pathlength through this tissue type. In the present study, we applied a MCS to 24 prefrontal channel positions of a typical fNIRS probe-set using three-dimensional segmented structural MRI data of 23 subjects. We simulated channel-wise NIR-light spatial absorbance maps to systematically quantify the impact of forehead anatomy on gray matter volumes traversed by the NIR-light before detection, i.e. the fNIRS sensitivity. Specifically, we studied the impact of individual and inter-channel anatomical variability of frontal sinus, SCD as well as sulcal geometry on these gray matter volumes.

## Results

### Spatial distribution of NIR-light absorption

The total absorbed energy from photon packages reaching the detector was 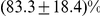 of emitted light. The mean number of NIR-light absorbing voxels within the six tissue types is displayed in Table 1. Of the voxels involved in NIR-light absorption within a channel cube, scalp 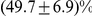 and skull 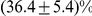 volumes outnumbered CSF 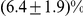, gray matter 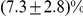, white matter 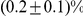 and air 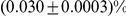. Similarily, the energy absorbed from photon packages reaching the detector differed between tissue types (scalp: 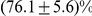, skull: 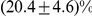, CSF: 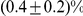, gray matter: 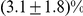, white matter: 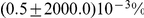, air: 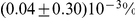). Absorbing gray matter had a mean volume of 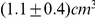 per channel.

**Table 1:** Tissue-wise volumes absorbing NIR-light.

CH#	air	scalp	skull	CSF	gray m.	white m.
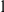	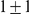	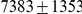	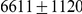	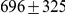	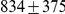	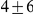
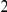	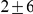	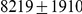	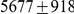	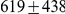	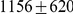	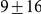
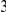	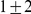	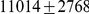	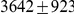	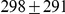	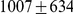	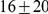
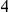	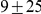	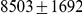	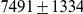	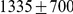	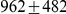	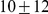
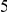	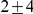	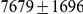	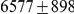	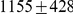	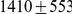	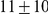
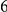	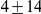	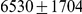	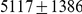	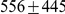	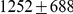	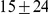
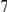	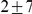	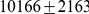	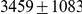	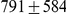	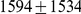	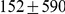
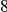	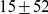	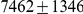	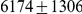	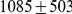	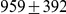	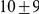
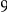	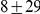	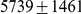	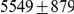	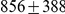	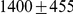	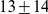
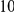	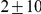	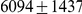	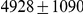	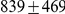	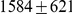	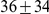
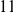	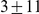	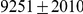	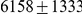	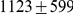	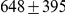	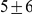
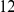	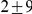	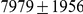	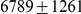	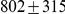	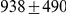	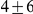
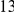	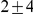	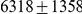	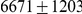	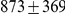	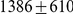	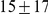
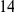	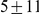	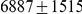	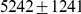	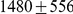	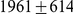	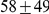
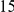	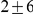	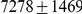	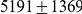	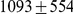	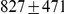	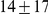
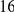	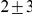	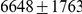	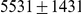	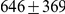	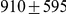	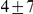
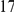	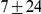	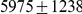	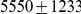	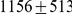	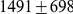	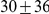
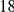	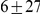	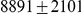	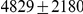	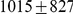	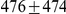	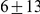
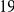	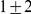	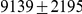	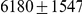	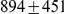	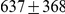	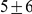
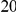	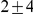	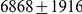	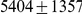	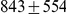	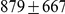	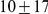
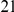	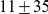	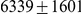	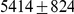	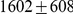	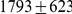	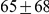
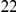	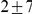	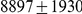	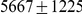	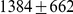	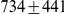	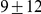
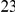	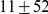	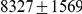	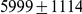	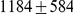	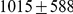	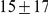
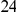	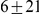	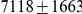	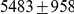	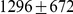	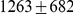	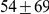
mean	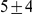	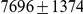	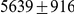	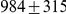	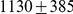	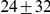

Mean channel- and tissue-wise number of voxels (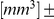 standard deviation) absorbing energy from simulated NIR-light photon packages before detection.

**Figure 1:**
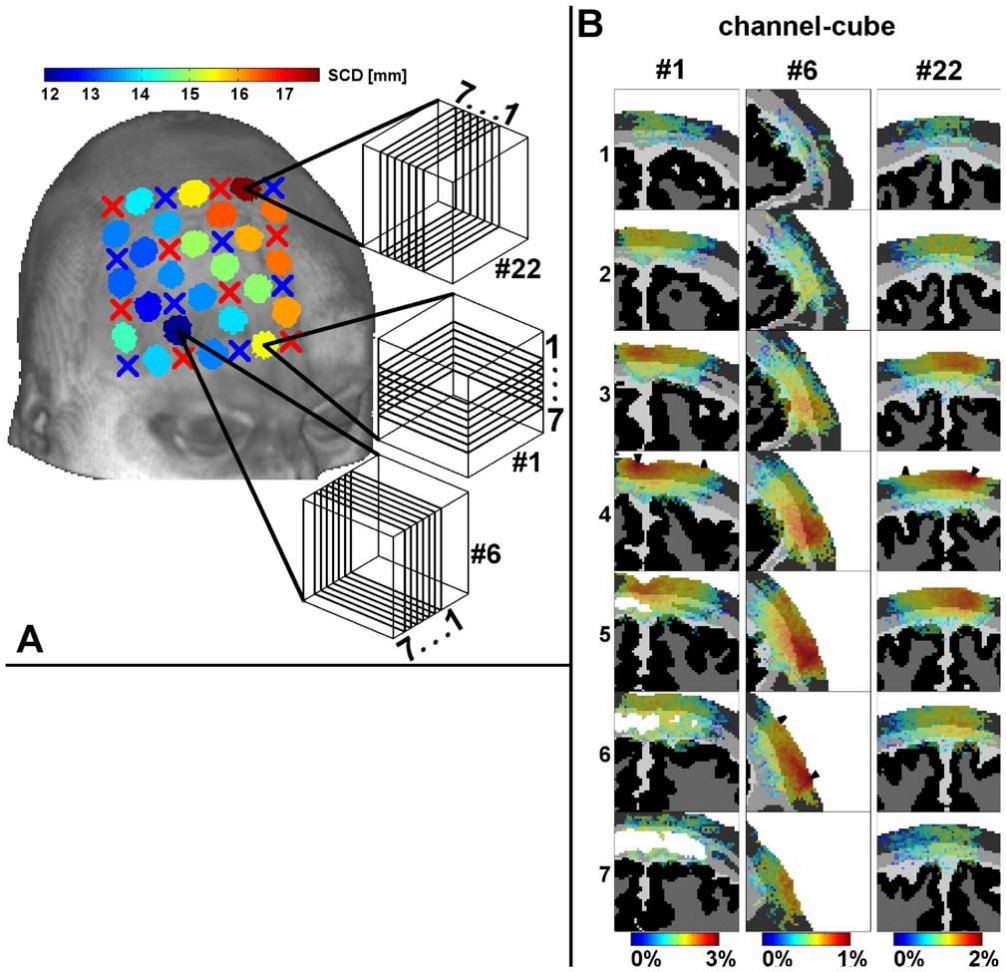
Illustration of SCD dependent light penetration. (A) The mean scalp to cortex distance (SCD) is indicated for the 24 channel positions showing an increase of SCD from right lateral prefontal cortex to posterior medial frontal cortex. (B) Three channels (subject #20) illustrate the spatial absorption distribution of NIR-light; Channel #22 (highest SCD), channel #6 (lowest SCD) and channel #1 (highest association with frontal sinus). The seven slices of each cube (cube: #1 transversal, #6 sagittal, #22 coronal) show the individual anatomically segmented images superimposed with the simulated absorption data. The logarithmic color scale indicates the percentage of energy absorbed in each voxel in relation to the total amount of energy absorbed in the whole channel cube.

Slices through three individual channel cubes (subject #20) exemplify the spatial distribution of different tissues and NIR-light absorbing voxels in (figure 1a, b). Slices of channel-cubes show an absorption pattern similar to an ellipsoid shape as slices move through the emitter-detector plane. The simulated individual absorption distributions reached gray matter in all channel-cube simulations, except in one case (subject #14, channel #18), and had similar spatial characteristics compared to the layer model simulations.

### Scalp-cortex distance and NIR-light absorption

The mean SCD over all channels and subjects was 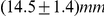. Mean SCD of NIRS-channels was between 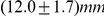 (channel #6) and 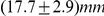 (channel #22). Channel #1 located over the frontal sinus had a mean SCD of 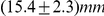. The mean SCD for each NIRS-channel (displayed in figure 1a) was negatively correlated with the corresponding simulated 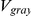 (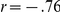, 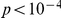). NIRS-channels with high mean SCD had significantly smaller 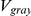 compared to channels with low mean SCD (median split; 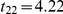, 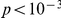; mean difference: 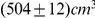). Similarily, between subjects the mean SCD (over channels) also significantly impacted the simulated 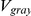 absorbing NIRS-light (correlation: 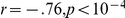; median split: 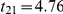, 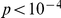; mean difference: 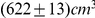). Note, that 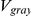 is highly correlated with the partial pathlength of NIR-light through gray matter tissue (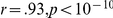). Furthermore, the circumference of the individual heads (mean: 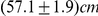 was positively correlated with the mean SCD (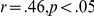) and negatively correlated with the mean 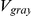 (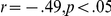) of the subjects. The correlation between inion-nasion distance with SCD and 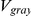, respectively, had the same sign of coefficient, but was not significant.

**Figure 2:**
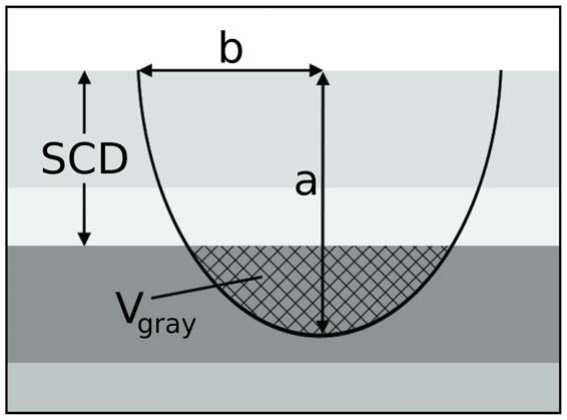
Illustration of the ellipsoid model used to estimate the volume 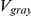
 **of light absorbing gray matter as a function of the scalp to cortex distance (SCD).** 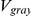 within the rotational ellipsoid calotte is solely dependent on the two length parameters 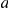 and 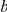, whereby 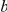 is half the optode distance (1.5 cm) and 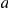 is a measure for the depth of light penetration.

Based on the assumption of a rotational ellipsoid traverse of NIR-light through the tissue [6], we derived a function describing the relationship between SCD and 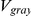. Here, 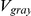 is represented by the ellipsoid calotte and 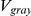 as a function of SCD is solely dependent on the ellipsoid parameter 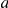 (see scheme of figure 2). 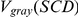 can thus be calculated by:
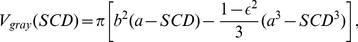
(3)where 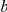 is the ellipsoid radius at the optode level and 
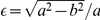
 is the ellipsoid function.

**Figure 3:**
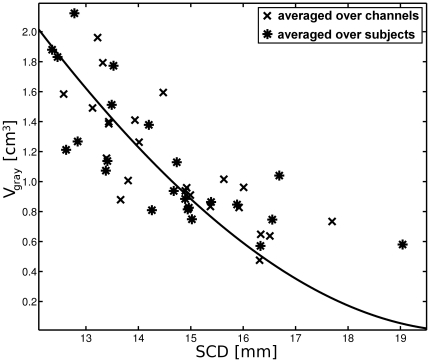
The relationship between scalp to cortex distance (SCD) and light absorbing gray matter volume 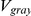
 **averaged over channels (crosses) or averaged over subjects (asterisks).** In order to define a curve (continuous line) describing this relationship, we fit the depth parameter 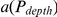 from the analytical expression for 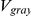 to the data, whereby 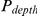 is the percentage of light absorption beyond a certain depth according to figure 4. For 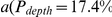 the best fit (least square) of the ellipsoid is achieved, which corresponds to 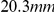 referred to as the relevant penetration depth 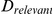.

**Figure 4:**
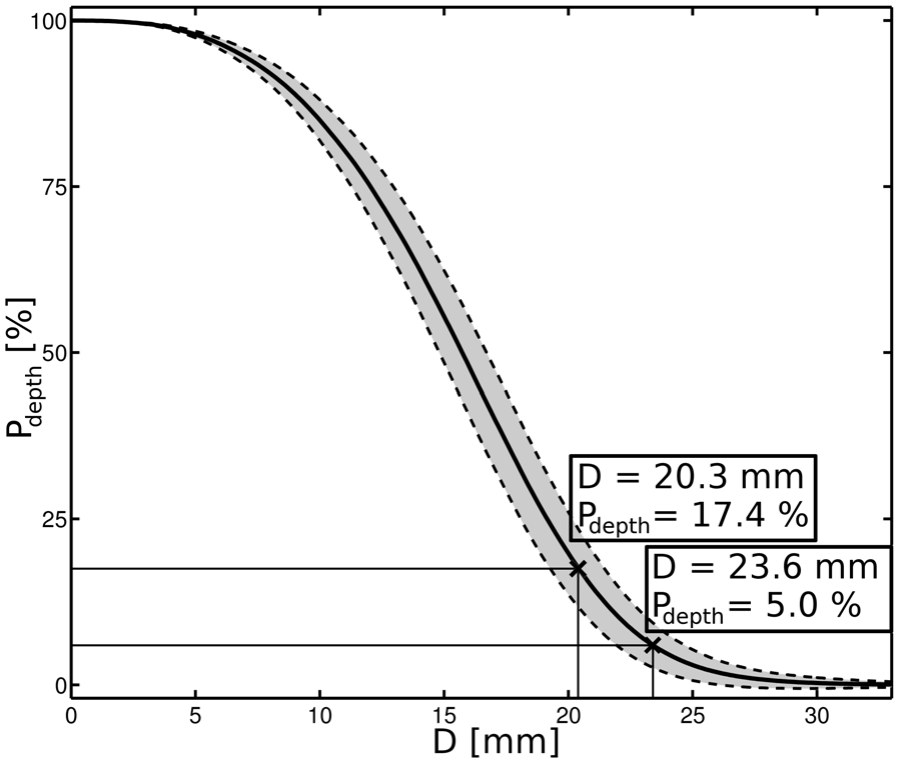
The cumulative absorption distribution within all channel-cubes shows the integral of the distribution of light absorbing voxels at a given distance 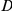
 **to the reference point between the respective optodes on the head surface.** The shaded area indicates SD. 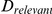 and the mean penetration depth of the 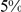 most distal reaching pathways are indicated.

The parameter 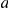 is a function of the cumulative probability distribution (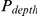) of the spatial distance of absorbing voxels within the channel-cubes (figure 3). The cumulative absorption distribution with increasing penetration depth of all channel-cubes is shown in (figure 4). We used these distributions to gain a value of 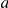 producing the best fit of the function 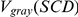 to the data, which was obtained for 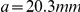 (i.e., the mean distance of the 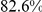 most proximal absorbing voxels calculated over all subjects and channels). This value of 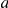 generating a rotational ellipsoid best describing the traversed depth of all channels and subjects we refer to as “relevant” penetration depth (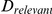). The 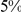 (arbitrary threshold) most distal reaching pathways have a mean penetration depth of 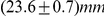.

**Figure 5:**
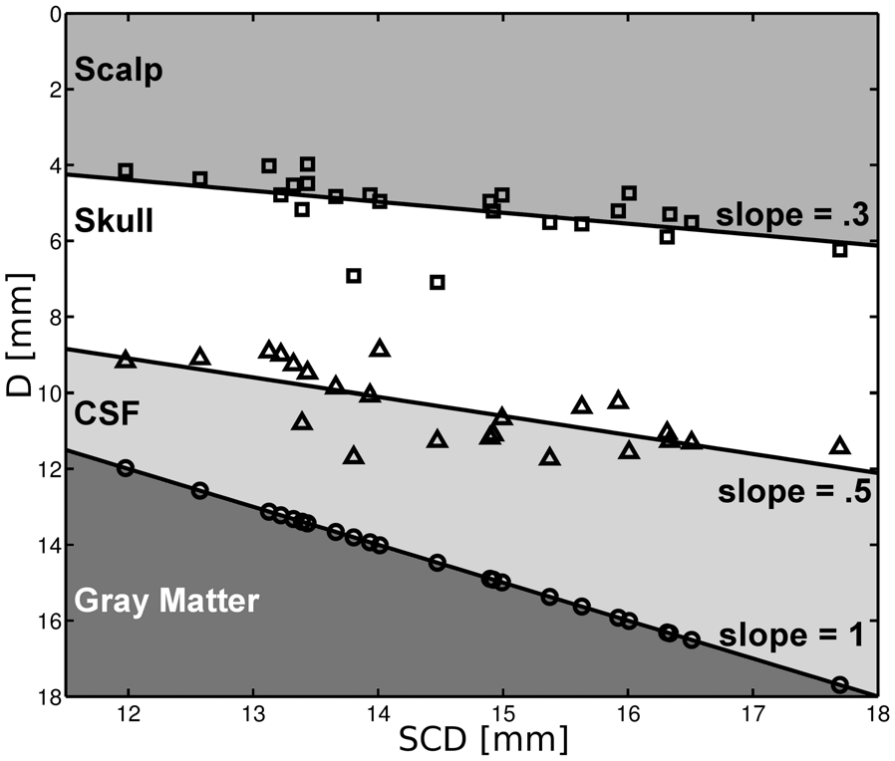
Scalp to skull distance (squares), scalp to CSF distance (triangles) and scalp to cortex distance (SCD, circles) are plotted against SCD to illustrate the proportional change of tissue types with increasing SCD. The smallest increase of thickness is observed for the skull layer (slope 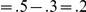), whereas the increase in CSF layer thickness with increasing SCD is twice as steep (slope 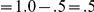). Note, that the 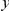-axis is flipped, thus decreasing lines have positive slopes.

**Figure 6:**
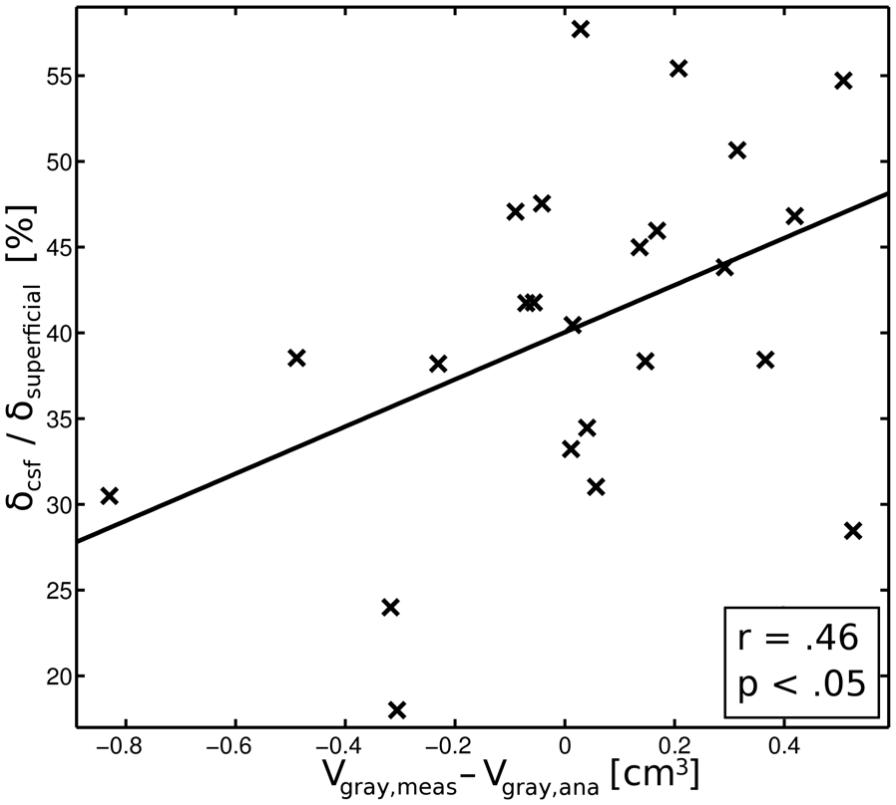
The ratio between the CSF layer thickness 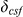
 **and the thickness of the superficial tissue layer** 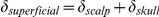 **plotted against the difference of the simulated light absorbing gray matter volume** 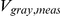 **and the analytically determined gray matter volume** 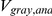 **(see** **figure 3** **).** The significant positive correlation indicates, that the simple ellipsoid model underestimates  when the CSF layer is relatively thick compared to the superficial tissue layer.

However, since mere distance from scalp to cortex may not fully explain the variance of 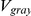, we investigated, if (1) scalp, skull and CSF, respectively, and (2) SCD-dependent proportional changes in tissue type compositions would underlie the profound reduction in 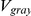 as SCD increases. First, we used a stepwise multiple linear regression of 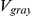 and the tissue type layer thickness as independent variables. Layer thickness of skull (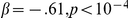), scalp (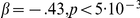) as well as CSF (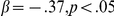) layer thickness significantly predicted 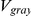. Second, we plotted the mean depth distribution of scalp, scull, CSF and gray matter tissue against SCD. The linear increase in CSF layer thickness with increasing SCD had a slope twice as steep compared to scalp and skull (figure 5). Previously, the presence of low scattering CSF has been shown to increase the partial pathlength through brain tissue compared to a head model with the same SCD but lacking CSF [18]. Therefore, the ellipsoid callote model as a function describing the relationship between SCD and 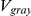 (figure 6) may be affected by the overproportional increase in CSF layer thickness 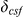 with increasing SCD. To test this, we correlated the difference between simulated (MCS) and analytically determined (ellipsoid function) 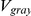 with the proportional ratio of CSF/(scalp+skull) layer thickness. These variables were significantly correlated (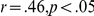; figure 6). Thus, the overproportional increase in CSF may attenuate the reduction of 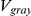 with increasing SCD.

### Frontal sinus and NIR-light absorption

In order to investigate the feasibility of fNIRS measuring functional hemodynamics within neural structures behind the frontal sinus, we assessed the impact of its volume on the NIR-light penetration depth as well as 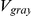 generated by the MCS.

Derived from the relationship between cumulative absorption distribution and penetration depth, we assessed the distance of the 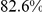 of absorbing voxels for each channel and subject to relate this distance (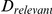) to the volume of air-filled frontal sinus traversed by the simulated photon packages. In channel #1 these variables were correlated (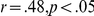); i.e. the larger the frontal sinus the deeper the penetration.

However, frontal sinus volume was negatively correlated with the simulated 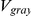 in channel #1 (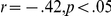). The correlation of the sum of traversed frontal sinus volumes of all respective channels (#1, #2, #4) with the sum of 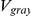 of these channels was even more pronounced (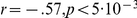;. Subjects with relatively large frontal sinus volume (sum of channel #1, #2, #4) showed a 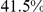 reduction in 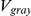 compared to subjects with low or no frontal sinus volume (median split; independent samples t-test: 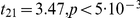; mean difference in 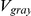: ). The individual head circumference was positively correlated with the sum of traversed frontal sinus volumes of channel #1, #2 and #4 (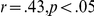).

### Gyrification and NIR-light absorption

**Figure 7:**
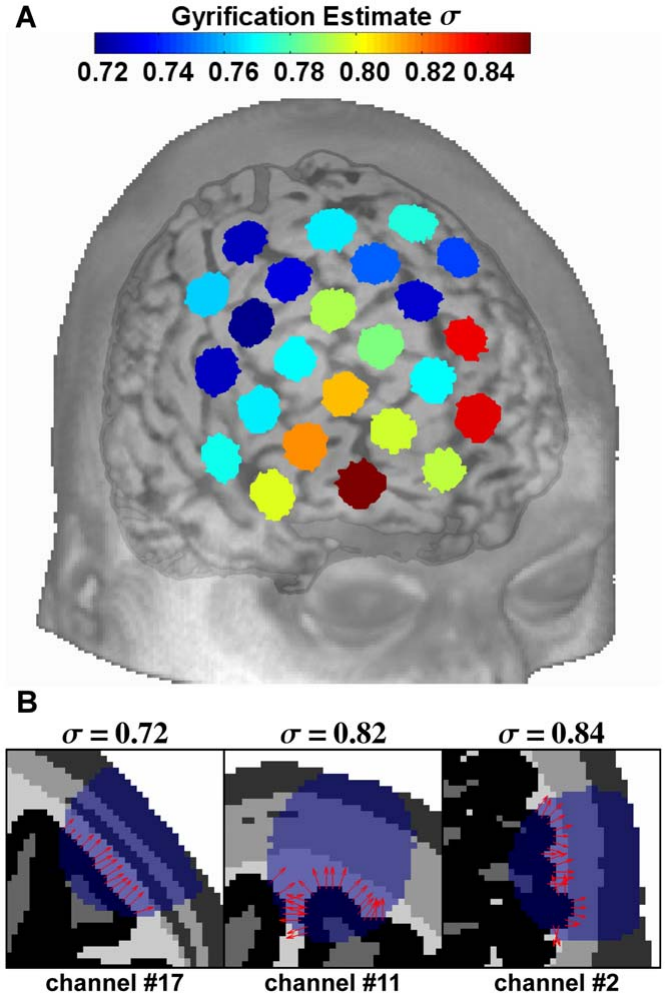
Illustration of gyrification analysis. (A) The mean gyrification estimate over all subjects is displayed on the averaged normalized probe-set. Conclusively, channels (#4 and #11) over the interhemispheric sulcus as well as channel #2, which probably covers parts of the middle frontal gyrus, showed the highest gyrification. (B) Three expamples illustrate the estimation of the gyrification magnitude of the cortex surface, i.e. the length of the standard deviation vectors (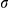) of the normal vectors (red arrows). These vectors were attached to the gray matter boundary to CSF within an ellipsoid with a depth parameter 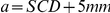. Individual slices from channel #17 (lowest gyrification), channel #11 (interhemispheric sulcus) and channel #2 (highest gyrification) with the respective ellipsoid slices (blue) are shown.

To investigate the potential influence of gyrification and sulcal geometry we calculated the mean standard deviation of gray matter surface vectors in 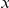, 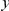, 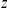 direction, respectively. The lenght of the resulting standard deviation vector indicated the magnitude of gyrification. We selected all gray matter surface voxels within ellipsoid volumes, which were individually selected by 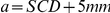. Three examples of gray matter surface vector constellations, ellipsoid slices and the corresponding standard deviations are given in (figure 7B). The gyrification estimates for each fNIRS channel (averaged over subjects) are shown in (figure 7A). Channel #4 and #11 located over the interhemispheric sulcus as well as channel #2 (probably over the orbital part of the middle frontal gyrus) showed increased gyrification. The mean gyrification estimate per channel was not correlated with the corresponding mean 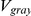 (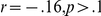).

## Discussion

In the present study, we implemented a Monte Carlo simulation of NIR-light traversing individual forehead tissue compositions. We investigated the impact of individual and inter-channel variability in anatomy on the tissue-specific distribution of NIR-light absorption from emitter to detector. The present study replicates two previous findings: (1) While NIR-light traverses biological tissues of the head (and is partly absorbed),the average pathway of the light and the average absorption distribution are best described by an elliptical curve [6] [16]. (2) Penetration depth of NIR-light (of about 2 cm to 3 cm at 3 cm interoptode distance) is negatively correlated with SCD, and SCD increases from right lateral to medial PFC [6].

Moreover, this study shows that (3) not only penetration depth, but also the simulated 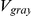 is highly negatively correlated with SCD. (4) While NIR-light absorption within skull and scalp mainly underlie the reduction of 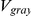 with increasing SCD, the low scattering CSF layer becomes overproportionally thick as SCD increases, thereby attenuating the 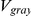 reduction. (5) Penetration depth is larger for fNIRS channels located over air-filled frontal sinus, but 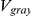 is significantly reduced. (6) The magnitude of gyrification has no significant impact on 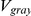.

These findings are discussed with regard to previous studies and their implications for fNIRS as a functional neuroimaging technique in human neuroscience and clinical research. MCS have repeatedly been used to provide numerical solutions for the forward problem of photon migration in highly scattering biological tissues [16], [18], [20], [21] to infer the NIR-light migration within head and tissue models. Another approch is to map NIR-light pathways using the spatial distribution of correlations between functional hemodynamic responses using simultaneous fNIRS and fMRI [6]. Our findings are in line with these previous studies, which were (relatively) consistent regarding shape (banana, ellipsoid) of NIR-light pathways and penetration depth estimates (about 2 cm to 3 cm). Complementing these studies we investigated a relatively large number (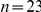) of individual segmented MRI data-sets to assess the impact of individual anatomy on NIR-light pathways and tissue-specific absorption profiles.

By comparing the spatial sensitivity profile of simulated NIR-light between a virtual head phantom (132 averaged T1 weighted MR images, segmented) and a simplified layered slab model [25] with uniform superficial tissue layer thickness, Kawaguchi et al. (2007) [26] showed that the uneven distribution of superficial tissue layer thickness in the head phantom impacts the mapping of simulated hemodynamics in the temporal area. While our study investigated frontal and not temporal areas, our results confirm and extend these findings by simulating the NIR-light penetration and absorption individually considering anatomical parameters for a large number of subjects. Moreover, we suggest, that a rotational ellipsoid of NIR-light absorption, that reaches to a variable degree (e.g. depending on SCD) into gray matter (see figure 4 and 5, equation 3) might be an illustrative explanation for this result.

Although biological tissues absorb relatively little of the NIR-light, the exponential attenuation of light intensity along a given pathway explains the differences in tissue-wise absorption (scalp and bone: 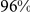, gray matter: 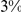). The total energy absorbed from photon packages reaching the detector amounts to 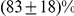 leaving some variability in absorption, i.e. fNIRS signal changes due to a functional increase of oxygenated and decrease of deoxygenated hemoglobin, respectively. However, fNIRS sensitivity is dependent on the traversed volume of gray matter in which this absorption change occurs. Since several sources of error variance may be part of the fNIRS-signal (e.g. extracranial blood flow, motor-artefacts, blood pressure), 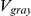 might be critically small in some subjects and/or channels associated with large SCD. We found, that especially skull layer thickness (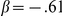) represents a good predictor for 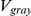 reduction. Previously, a layer model with thicker skull layer instead of low scattering CSF has been shown to decrease partial optical pathlength in brain tissue compared to a model with the same SCD including CSF [18]. We show, that skull, scalp as well as CSF contribute to the reduction of 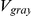 in healthy adult subjects. However, an overproportional CSF layer thickness may attenuate the reduction, thus, not the mere SCD but the partial pathlengths in specific tissue types have to be considered for the estimation of 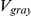 or anatomy-dependent fNIRS-sensitivity.

Since structural MRI might not be available for most participants of fNIRS studies, we suggest to consider head circumference as a practical predictor of SCD and 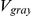, thus, the fNIRS sensitivity. Head circumference could easily be accounted for when introduced as a covariate of fNIRS data or could be matched when comparing groups. Also, large differences in head circumference may affect the reliability of the assignment and functional measurement of putative neural structures spatially associated with a certain fNIRS channel. In this regard, mean head circumference has been shown to significantly differ between men with 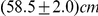 and women witch 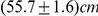 [27], which may play an important role in the light of our findings.

A possible explanation for the frontal sinus to decrease 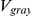 could be the presence of an air-filled cavity, which represents an extreme case of differential tissue layer composition. Regarding the transition of the light between skull and CSF (transition 1, normal case) on the one hand and between skull and air (transition 2, only possible at frontal sinus) on the other hand the most striking difference is the ratio of refractive indices (table 2), i.e. difference of optical impedance is higher for transition 2 than for transition 1. Using Snell's Law and equation (9) and considering total internal reflection the probability for the light to be reflected at transition 1 (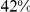) and transition 2 (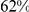) was calculated. Thus, the light reflection increased by ca. 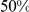 at the frontal sinus (transition 2) compared to the transition (transition 1) without frontal sinus. By means of Monte Carlo simulation Seunghwan et al. (2005) [28] investigated the effect of an refractive index mismatch between tissue and CSF and found that such a mismatch can lead to changes in measured light intensity.

**Table 2:** Optical coefficients.

	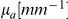	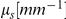	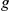	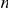
Air	0	0	0	1.00
Scalp	0.016	19.0	0.9	1.60
Skull	0.018	16.0	0.9	1.56
CSF	0.004	0.3	0	1.33
Gray matter	0.090	21.5	0.9	1.40
White matter	0.090	38.4	0.9	1.47

Optical coefficients of different tissues used for the MCS. Refraction coefficients were taken from [39] except for scalp: [40]. Scatter, absorption and anisotropy coefficients of skin and bone were from [17]. Since coefficient values for gray and white matter differed between sources [41]–[43], the respective values were averaged. The light wavelengths used for assessing these coefficients were between 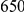 nm and 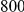 nm.

The air-filled frontal sinus mostly covers parts of the OFC. As the OFC has been shown to be centrally involved in decision-making and the processing of the subjective evaluation of reward, emotional, social, and sensory information [29], future fNIRS measurements will probably aim to further study its function. Our findings suggest, that a reduction in 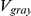, restricted or in extreme cases lack of fNIRS sensitivity due to large frontal sinus volume may have to be considered as sources of error variance in studies aiming to measure OFC activity. While we found traversed frontal sinus volume to be correlated with the head circumference (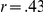), the morphology of the frontal sinus has been shown to be individually highly variable [12]. Future fNIRS studies aiming to measure the OFC may, thus, benefit from additional anatomical information of individual CT or MRI scans.

Although we found no significant impact of the gyrification estimate on the simulated 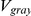 in prefrontal cortex, we cannot exclude the possibility that extreme cases of sulcal geometry as in the area of the fronto-temporal junction may affect fNIRS sensitivity.

Currently, the feasibility of fNIRS for single subject analyses of functional hemodynamics to derive an individual psychiatric diagnosis of e.g. depression, biploar disorder or schizophrenia is controversially debated [30]. Here, our findings suggest cautious interpretation of fNIRS signals on a single subject basis, because individual anatomy regarding SCD and frontal sinus volume may profoundly impact fNIRS sensitivity. Aforementioned psychiatric illnesses themselves are associated with anatomical changes such as a reduction in gray matter layer thickness [31]–[33]. Also, the impact of cortical atrophy and increased CSF volumes after long-term alcohol abuse or in patients with Alzheimer's disease on fNIRS sensitivity has not been investigated yet. Because the participating subjects were young and healthy, this question could not be addressed in the present study. Thus, further functional measurements and simulation studies of anatomical factors modulating fNIRS signals are necessary to quantify the influence anatomical differences exert on fNIRS sensitivity.

## Materials and Methods

### Participants

A total of 23 adult healthy subjects (12 male) with a mean age of 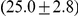 years including 4 left-handed subjects (2 male) were included in the study. One subject (#10) was excluded due to noisy structural MRI. To exclude any history of Axis I or II pathology, neurological disorders or psychoactive medication all subjects were screened by questionnaires based on DSM-IV criteria for Axis I mental disorders. All participants gave written informed consent. The study was in accordance with the latest version of the Declaration of Helsinki and approved by the Ethics Committee of the University of Wuerzburg.

### fNIRS optode positions

**Figure 8:**
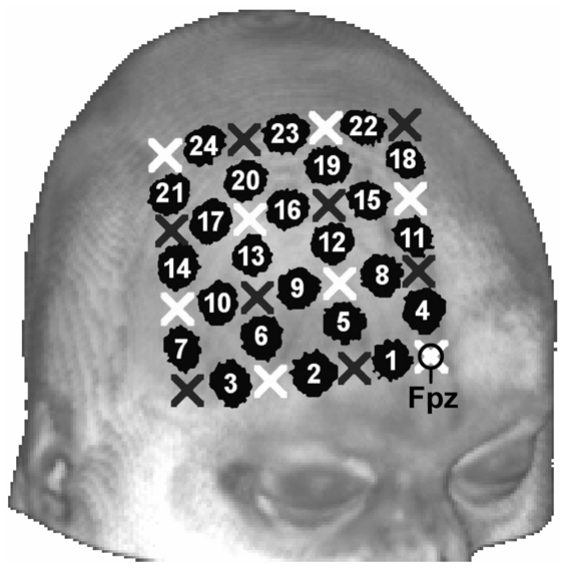
Normalized and averaged fNIRS channel positions on the right lateral forehead. Numbers indicate channels, bright and dark crosses indicate NIR-light emitter and detector positions, respectively. By using the International EEG 10–20 system the emitter of channel #1 and #4, respectively, was positioned on the Fpz marker point while the bottom row of channels was placed on a line between Fpz and F8.

We precisely defined optode locations using a typical 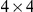 fNIRS probe arrangement (ETG-4000, Hitachi Medical Systems, Japan), which was positioned over the right prefrontal cortex. A set of 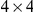 optodes, which correspond to 24 fNIRS recording channels each consisting of an NIR-light emitter-detector pair, was used in the present study. The probe set was placed in respect to the Fpz surface marker of the International EEG 10–20 system (figure 8). Specifically, the light emitter of the NIRS-channel #1 and #4, respectively, was placed on Fpz while the bottom row of NIRS optodes of the probe set was located on a line from Fpz to F8. A previous pilot fNIRS-fMRI measurement confirmed feasibility of identification of precise optode positions as probe-holder of each optode and/or slight skin indentation by optode tips were visible in the structural image. This positional information defined native spatial coordinates of the 24 NIRS channels in each subject using the MRIcro software [34]. The channel coordinates between two optodes were normalized to MNI-space using SPM8 normalization routines. The normalized coordinates were then spatially averaged over all subjects and indicated by blobs of 5 voxels diameter using MRIcro. The channels #1, #2 and #4 are mostly associated with parts of the frontal sinus with most volume underneath channel #1. The normalized and averaged probe set is displayed in figure 8.

### Head circumferences

The head circumference of each subject was measured using the individual Fpz marker point (International 10–20 system) and inion head surface position. From these two points the head circumference was assessed by a mearsuring tape.

### Individual tissue models

Structural MRI images were acquired using a 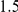 Tesla Siemens MAGNETOM Avanto syngo (MR B17) scanner. 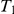-weighted structural images were recorded with a spatial resolution of 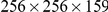 voxels with a voxel size of 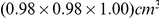 using 3D magnetization prepared rapid gradient echo (MPRAGE) sequences with a repetition time (TR) of 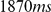 and an echo time (TE) of 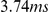.

Automatic anatomical tissue segmentation was performed using the SPM8 tool New Segment, that is an extension of the default unfied segmentation [35]. The new segmentation tool was used because it is possible to obtain probability images for scalp and skull and not only for background ( = air), CSF, gray and white matter. The algorithm classfies different tissue types by using their respective intensity distribution and a segmented template. Because voxel intensities are used for tissue classification the structural images must be corrected for modulations of intensity caused by noise. In low intensity regions (such as frontal sinus) the noise after segmentation was suppressed by smoothing the individual probability images with a 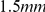 Gaussian kernel. These probability images are used to create an individual tissue model for each subject. To summarize the results of the segmentation table 3 and figure 7 give an overview of the tissue layer thickness of scalp, skull and CSF.

**Table 3:** Thickness of scalp, skull and CSF layer.

CH#	 [mm]	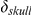 [mm]	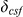 [mm]
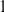	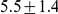	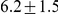	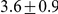
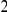	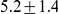	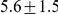	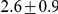
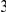	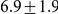	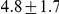	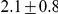
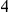	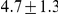	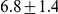	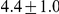
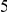	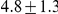	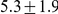	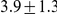
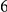	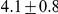	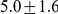	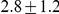
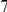	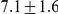	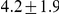	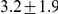
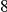	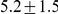	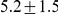	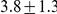
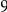	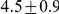	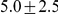	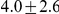
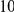	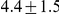	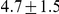	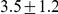
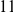	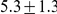	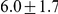	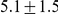
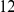	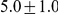	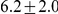	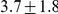
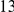	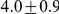	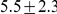	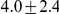
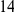	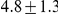	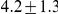	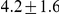
	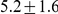	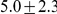	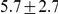
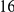	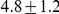	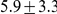	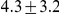
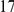	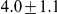	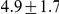	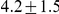
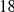	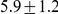	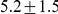	
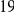	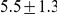	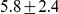	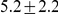
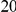	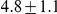	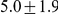	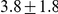
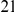	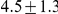	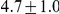	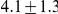
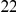	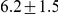	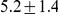	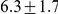
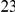	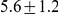	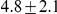	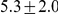
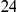	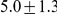	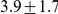	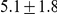

Layer thickness of scalp (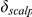), skull (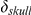) and CSF (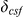) averaged over subjects with standard deviation.

However, the segmentation may have been improved by using multi-spectral data, e.g. additonal 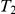 weigthed images [35]. Unfortunately, such additional structural information was not available in the current study.

### Monte Carlo simulation

The purpose of the Monte Carlo simulation (MCS) implemented in this study is to describe the interaction of light within different tissue types in individually segmented human heads. We thereby characterize the spatial distribution of absorbed energy in voxels along NIR-light pathways from emitter to detector. As a guideline for implementation served the MCS by [36]. The source code of the MCS was written in C++ and compiled with MinGW 4.4.0. Data preparation and analyses of simulation data were performed using custom software written in MATLAB 7.9 (The MathWorks, Natick, MA, USA). Statistical analyses were performed using SPSS 17.0 (SPSS Inc., Chicago, IL, USA).

### Photon packages

The MCS consecutively and perpendicularly launches photon packages from the emitter into the individual foreheads. For the packages of photons an energy amount referred to as *weight* 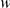 was defined and initialized with 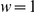. These packages moved through the native space of the segmented brains, which was described by a three dimensional vector 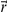 in carthesian coordinates [mm]. The direction of the photon package migration was described by a unit vector 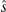, that holds the three direction cosines in its components. A positional change from 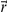 to 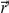 with a given step size 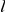 was given by:
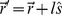
(4)In order to reduce computational load of the channel-wise MCS, a cube of 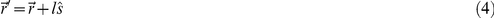 voxels was selected comprising the emitter-detector positions on the scalp and segmented tissue underneath. In the following, we refer to these cubic volumes of interest as channel-cubes.

### Probabilistic light interactions

Because the absorption and scattering coefficient 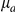 and 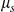 were defined as rates, the probability of a photon interacting after a step size 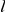 follows an exponential decay and the normalized probability distribution 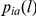 was calculated by:
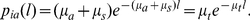
(5)After the photon traveled this random step size 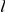, the current weight was reduced due to absorption:
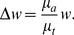
(6)The scattering caused a direction change from 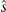 to 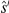. To describe this process a coordinate system was created, in which the unit vector of the 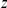-axis was equal to 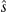. Thereafter, the new direction 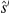 was specified by the two angles 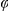 and 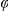:
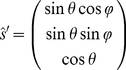
(7)Due to the cylindrical symmetry of the scattering process, the angle 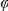 is uniformly distributed between 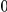 and 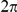. The probability distribution of the angle 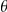 is given by the *Henyey-Greenstein* phase function [37]:
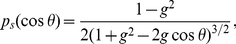
(8)where the parameter 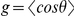 quantifies the anisotropy of the scattering. For values of 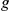 close to 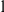 as in biological tissues (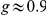) forward scattering strongly prevails.

### NIR-light reflection and refraction

Depending on the difference in refractive indices (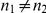) between two tissues, a photon hitting the phase transition with an incident angle 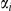 is either reflected with the angle 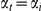 or transmitted with a refraction angle, which was calculated using *Snell's law* (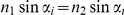). To obtain the incident angle of the photon relative to a boundary between two tissues, a normal vector was attached to all voxels of this boundary. These normal vectors were defined as unit vectors perpendicular to the surface while considering 3 to 6 neighboring surface voxels in two perpendicular directions, respectively.

The acute incident angle of a photon hitting a surface was calculated by the dot product between the photon direction 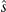 and the normal vector 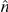 at the corresponding voxel: 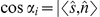. By including the refraction angle 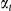 from Snell's law, the reflection probability 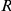 was obtained using the *Fresnel* equations under the assumption of equally distributed polarization directions:
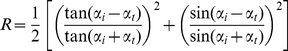
(9)The probability 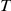 that a photon is refracted was: 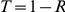.

### Random processes

The step size 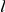 with its probability distribution from equation (5) was given by
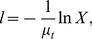
(10)where 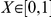 is an equally distributed pseudorandom number. Wheather a photon hitting a phase transition was reflected or transmitted was determined by the test 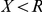 (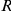 from equation (9)). If the test is true, the photon was reflected, otherwise it was transmitted. The cosine of the scattering angle 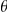 was given by:
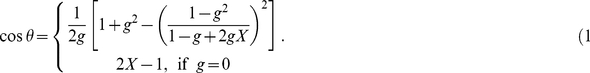
(11)using the distribution from equation (8).

### Measured quantities

We simulated the spatial distribution of voxel-wise absorbed energy of NIR-light photon packages reaching the detector within the channel-cubes. Each weight loss 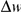 at a location 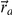 was registered to the corresponding location in the cubic voxel-space (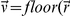)). By using the tissue type information of each voxel, the magnitude as well as the tissue volume contributing to 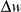 were calculated. Location data of all simulated light-matter interactions were collected and used to generate NIR-light pathways reaching the detector. Then, the partial pathlength through gray matter was calculated for each photon package. To quantify the penetration depth the distances 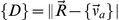 between each voxel contributing to 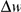 and a reference point 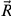 centered between respective channel optodes on the scalp were calculated.

### MCS configuration

Optical properties of each tissue type were described by four coefficients taken from the literature: Absorption coefficient 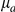, scattering coefficient 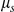, anisotropic parameter 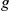 and refraction index 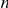. The coefficient values are listed in table 2.

Depending on 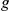 the coefficient 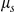 is reduced, and deduced from the diffusion approximation of the radiative transport equation 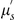 is defined as:
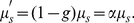
(12)


**Figure 9:**
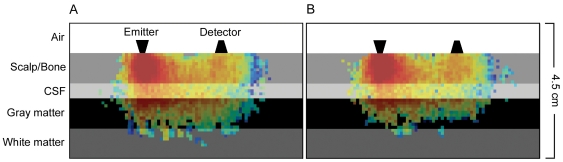
Slice through optode plane of a simple tissue layer model, that was implemented to validate the configuration. The absorption intensity is logarithmically scaled showing the distribution of high (red) and low (blue) absorption. (A) Absorption distribution using scattering coefficients 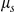 and anisotropic scattering (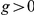). (B) Absorption distribution using reduced scattering coefficients 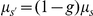 in conjunction with isotropic scattering (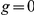 for all tissues). The two distributions overlap more than 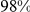, while the simulation under the conditions of (B) runs around six times faster.

For 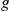 close to 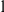 the coefficient 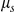 is strongly reduced. Thereby, the mean step size 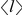 increases by the factor 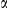 reducing the number of interactions. In order to reduce MCS calculation time by the factor 6, we used 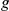-dependent 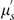, but instead simulated isotropic (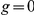) instead of anisotropic (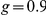) scattering. Another advantage of this approach is that the calculation of the scattering angle 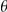 following equation (8) is faster and not prone to errors as described by [38]. A possible impact of this procedure on the distribution of absorption was ruled out by applying the simulation to a simple layer model (figure 9a, b). Due to variability of absorption coefficients (in the literature) we used the simple tissue layer model to investigate the impact of coefficent value variations on the simulated absorption pattern. We found that the absorption distributions of a run with original coefficients and a run with scattering coefficients decreased by factor 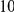 still overlapped by 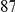
%. Thus, the simulation was stable under coefficient variations.

To estimate how many photons are required for the MCS to obtain stable absorption distributions, the MCS was conducted for channel #1 and #9 of two subjects with increasing number of emitted photon packages 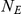. We used seven equal photon package number increments (from 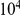 to 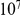) and calculated the overlap of the normalized absorptions between two runs. The absorption overlap comparing 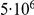 and 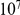 emitted photons was greater 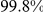, while approximately 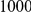 and 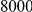, respectively, were detected. Therefore, we simulated each channel until 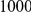 photons hit the detector. In total, we conducted simulations of 24 channels
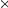
23 subjects
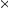
approximately 5 million photon packages 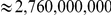 photon package simulations.

### Scalp-cortex distance

The SCD was individually assessed for each subject and NIRS channel. We calculated the radial distance between the reference point on the head surface and the closest volume per mille of gray matter within a channel cube. The layer thickness of scalp, skull and CSF layers (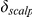, 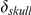, 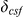) were obtained analogously.
